# Optimization of Modified Atmosphere Packaging for Sheep’s Milk Semi-Hard Cheese Wedges during Refrigerated Storage: Physicochemical and Sensory Properties

**DOI:** 10.3390/foods12040849

**Published:** 2023-02-16

**Authors:** Marta Albisu, Sonia Nieto, Olaia Martínez, María Ángeles Bustamante, Luis Javier R. Barron, Ana Isabel Nájera

**Affiliations:** 1 Lactiker Research Group, Faculty of Pharmacy, Universidad del País Vasco/Euskal Herriko Unibertsitatea, 01006 Vitoria-Gasteiz, Álava, Spain; 2 Efficient and Sustainable Processes Department, Bizkaia Technology Park, AZTI, P.O. Box 609, 48160 Derio, Bizkaia, Spain; 3 Texture Analysis Laboratory, G3S Research Group, Faculty of Pharmacy, Universidad del País Vasco/Euskal Herriko Unibertsitatea, 01006 Vitoria-Gasteiz, Álava, Spain

**Keywords:** ripened cheese preservation, modified atmosphere packaging, vacuum, cheese wedges, sensory properties

## Abstract

Modified atmosphere packaging (MAP) has become a good potential strategy to retain quality throughout the shelf life of perishable foods. The aim of this work was to evaluate different packaging atmospheres on semi-hard protected designation of origin Idiazabal cheese wedges. Six different packaging treatments (air, vacuum, and CO_2_/N_2_ gas mixtures in the ratio of 20/80, 50/50, 80/20, and 100/0% *v/v*, respectively) were studied. Changes in gas headspace composition, cheese gross composition, weight loss, pH, acidity, colour, and textural and sensory properties were investigated during 56 days of refrigerated storage at 5 ± 1 °C. MAP was the most effective preserving technique compared to air- and vacuum-packaging treatments. The cheese characteristics with the greatest discriminating weight in the preservation techniques were paste appearance, holes, flavour, a* (redness) and b* (yellowness) colour parameters, and slope to hardness. Air-packaged cheeses, on 35 day, presented a mouldy flavour. Vacuum packaging affected paste appearance (greasy, plastic marks, and non-homogeneous colour) and holes (occluded and unnatural appearance) starting after 14 packaging days. MAP mixtures with CO_2_ concentration between 50/50 and 80/20% CO_2_/N_2_ (*v/v*) are recommended to ensure sensory quality and stability in the distribution of these raw sheep-milk cheese wedges.

## 1. Introduction

World cheese production in 2022 was approximately 22.08 million tonnes [1], where the European Union (EU) is the world’s most important cheese manufacturer, with a production of 10.4 million tonnes in 2021 [2]. The most widely produced cheeses are from cow’s milk, with continuous processing throughout the year, while small-ruminant cheese production is smaller and seasonal. The production of sheep’s milk cheeses has increased substantially in the Mediterranean countries. Spain is one of the largest producers in the EU, with 72.2 thousand tonnes in 2021 [3].

Given the seasonality of this cheese production, the herds spend a large part of the time grazing, which influences the quality of the milk and the regularity of cheese manufacture. Sheep-milk cheeses are highly appreciated by consumers, and this is partly because many of them are sold under quality brands such as protected designation of origin (PDO) or protected geographical indication (PGI) certifications [4]. Idiazabal PDO is a seasonal, pressed hard, and semi-hard raw-milk sheep’s cheese produced in the Basque Country (northern Spain). The milk-producing sheep must be of Latxa breed. The weight of Idiazabal PDO cheese is between 1–3 kg, with a minimum ripening time of two months [5]. These cheeses maintain acceptable sensory quality for a short period according to their brief manufacture season [6]. Therefore, it is extremely necessary to improve the preservation method in order to increase availability over a longer period without changing the sensory features. This traditionally made cheese is usually sold as a whole piece. However, there is an increasing consumer demand for portioned cheeses, and packaging is of great importance to ensure their quality and safety [7].

Shelf-life extension by packaging optimization has become a powerful strategy to improve marketing needs and reduce food waste or loss [8]. Different packaging systems have proven their usefulness to prolong the shelf life of cheeses and specifically to prevent several faults that may come with portioning. When combined with low-temperature storage, vacuum and modified atmosphere packaging (MAP) have effectively proven to be useful in cheese preservation [9], but the results depend on the cheese variety [10,11].

Vacuum packaging reduces oxidative damage and inhibits aerobic microorganisms’ growth. It might prevent dehydration and weight loss in cheeses as well as the incorporation of undesirable odours [12]. This preservation method has been successfully tested for hard and semi-hard cheeses [12,13,14]. However, negative effects have also been described, as this packaging provides anaerobic conditions and favours pathogens and moulds growth [15,16] as well as changes in cheese appearance [17,18].

MAP has been reported to extend the shelf life of cheeses, based on microbiological and sensory parameters, with certain gas mixtures (CO_2_ and N_2_) and for some types of non-pasteurized cheeses such as Crottin de Chavignol, Pasta Filata, and some mould-surface cheeses [19,20,21]. Carbon dioxide is the most important gas from a microbiological perspective because it inhibits the growth of spoilage bacteria such as aerobic Gram (−) and moulds and to a lesser extent Gram (+) bacteria and yeasts. On the other hand, N_2_ is used as a filler preventing package collapse. The gaseous composition of the atmosphere can change during storage time due to cheese breathing, biochemical reactions, and the diffusion of gases through the packaging material. A CO_2_ concentration between 20 and 60% (*v/v*) in the atmosphere is required for antimicrobial effect [9]. Usually, high concentrations of CO_2_ are used to suppress undesirable microbial growth, particularly in hard and semi-hard cheeses [21,22]. However, atmosphere design is more complex for raw-milk cheeses or those with starters added [23]. For these cheeses, CO_2_ concentration must be carefully adjusted and controlled; otherwise, the bacterial metabolism, enzymatic activities, lipid oxidation, and proteolysis involved in the development of cheese flavour may be modified, and off-flavours may appear during storage [10,13,21,24]. Moreover, this approach is of great interest for semi-hard cheeses presented in portion packs, as inner faces are exposed to atmospheres, and the best packaging options must be chosen in order to offer products with a longer durability to consumers. Preservation studies play a fundamental role in increasing the conservation time of portioned hard and semi-hard cheeses and can be of significant importance for the profitability and sustainability of the dairy sector [9].

Therefore, the aim of this work was to evaluate the influence of different types of packaging conditions—air, vacuum, and four different modified atmospheres—to see how they affect the quality of a sheep’s milk cheese. Physicochemical, colour, and textural properties and sensory quality of Idiazabal semi-hard cheese wedges with a maturity degree of three months were studied for a period of two months. The ultimate goal is to select which of the packaging options best preserves quality during the distribution stage of this type of cheese.

## 2. Materials and Methods

### 2.1. Cheese Collection, Packaging, and Sampling

A total of 60 raw sheep’s milk cheeses with three ripening months were purchased at a local dairy farm registered at the Idiazabal PDO Council. Cheeses were produced in different days, 24 h from each other, which constituted two batches. Idiazabal cheese, as it is produced with raw milk, needs a minimum of two ripening months to meet food safety and sensory requirements. Three ripening months were chosen, as it is then when the cheeses have already developed their typical sensory characteristics [5].

From the whole pieces of cheese, 360 wedges (175–200 g) were obtained. Cheese wedges were cut and immediately packed in commercial polyamide/polyethylene (20/70) pouches of 90 µ thickness (Merkapack, Vitoria, Spain). Pouches presented oxygen permeability of ≤80 cm^3^/m^2^ × bar × 24 h (75% HR), carbon dioxide permeability of ≤174 cm^3^/m^2^ × bar × 24 h (0% HR), and ≤2 g/m^2^ × bar × 24 h (85% HR) for water vapour. Cheese wedges were packaged under air, vacuum, and four different CO_2_/N_2_ gas mixtures: 20/80, 50/50, 80/20, and 100/0% *v/v*, (MAP1, MAP2, MAP3, and MAP4, respectively). Pouches were evacuated, flushed, and sealed using MAP equipment (Model EVT-450/20, Irimar, Lesaka, Spain) with gas injection. Carburos Metálicos-Grupo Air Products (Cornellá de Llobregat, Spain) supplied food-grade gases, and a binary gas hand-operated mixer model MM-2K N_2_/CO_2_ (Witt, Witten, Germany) was used. Two sets of samples were prepared and stored in a refrigerator at 5 ± 1 °C for 56 days.

Headspace gas composition, physicochemical, colour, and instrumental textural and sensory analyses were conducted on the cheese samples at 14, 21, 28, 35, 42, 49, and 56 storage days; the same analyses were carried out on the cheeses prior to packaging.

### 2.2. Headspace Gas Composition of Packed Cheese Wedges

Immediately after packaging in the laboratory, pouches were subjected to a visual inspection of the sealing area to check for possible failures. Gas composition was verified by means of a gas analyser, Oxybaby (Witten, Germany), at every starting point when supplying a new ratio of gases. On each sampling day, O_2_ and CO_2_ concentrations were checked again.

### 2.3. Physicochemical Analysis of Cheese

Cheese wedges were weighed before packaging and on each sampling day on an Adam balance (Milton Keynes, UK). Before the analyses, the samples were equilibrated at 17 ± 2 °C. Sample temperature was controlled with a penetration thermometer Testo model 104-IR in triplicate (Barcelona, Spain).

pH was determined, in quadruplicate, at different points along each wedge by means of a pH meter with a penetration electrode (Crison, Barcelona, Spain).

Titratable acidity was measured in duplicate according to ISO/TS 11869 [25] method for fermented milks, adapted according to the AOAC 920 method for cheeses [26], in which the volume of filtered aliquot was modified and expressed as g lactic acid/100 g cheese.

A Zeutec model 110-A100-1 infrared spectral analyser 2.0 (Rendsburg, Germany) was used to determine dry matter concentrations, total protein, and fat, previously calibrated using the application worxG2 software with a multiple linear regression (MLR) model. Measurements were performed in duplicate for each batch from a homogeneous fraction obtained from grating each cheese wedge after the removal of approximately 1 cm of rind.

### 2.4. Cheese Colour Measurement

A Minolta Chroma Meter CR-200 (Madrid, Spain) was used for colour measurement on one side of the cheese wedges in triplicate. CIELab values (lightness, L*; redness, a*; and yellowness, b*) were measured with the standard illuminant D65 and a visual angle of 10°.

The yellow index (Yi) colour expression [27] used by Romani et al. [13] and Favati et al. [17] was calculated. In addition, the yellowness index (Zi) parameter [28] reported by del Caro et al. [29] was also calculated.

$$\mathrm{Yi}=\frac{142.86b*}{L*}\;\;\;\;\;\mathrm{Zi}=100{(\frac{L*+16}{116}-\frac{b*}{200})}^{3}$$

### 2.5. Texture Profile Analysis of Cheese

**Figure 1:**
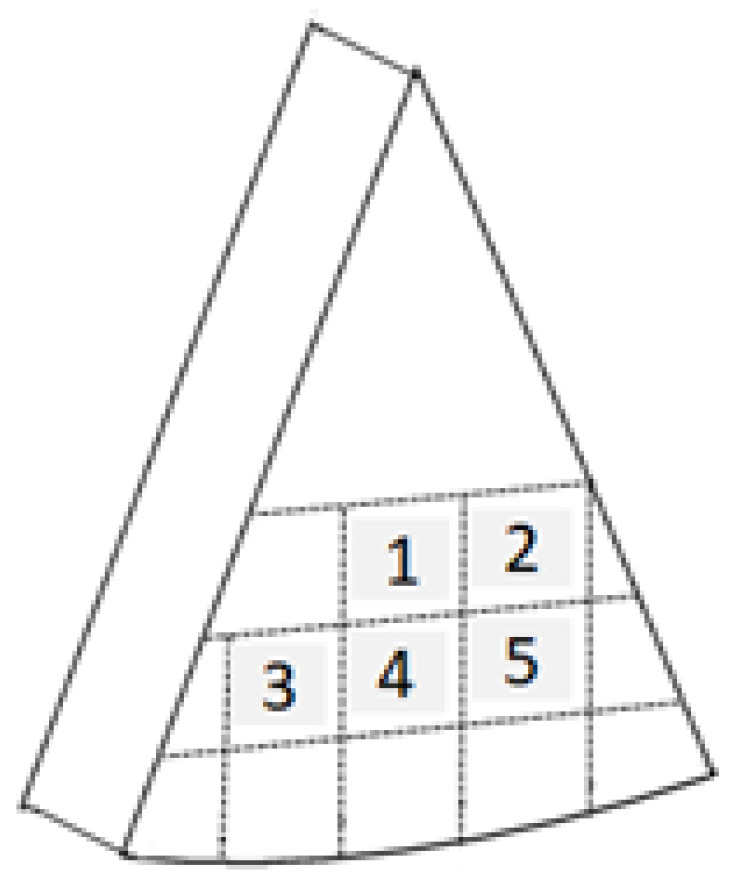
Scheme of the cube shaped samples used as repetitions for texture profile analysis within each slice obtained from cheese wedges.

Sample cubes were prepared as follows from each wedge: a 1 cm thick slice was removed from the rind in one lateral side of each wedge, and then, using a guide, three consecutive 1 cm thick slices were cut. Five cubes (1 cm × 1.25 cm × 1.25 cm) were obtained from each slice (Figure 1). These cubes were used as repetitions for texture profile analysis (TPA). Texture was assessed with a TA.XT2plus texture analyser (Stable Micro System, Surrey, UK) by means of TPA [30], with a 5 kg load cell. Two consecutive compression cycles at 15% were performed on cheese cubes, always with the narrower side upwards and using an aluminium cylindrical probe (diameter = 2.5 cm) at 1 mm/s. Cheeses were at 17 ± 2 °C during the assay. Texture Expert Exceed software was used for data processing.

The texture parameters studied were the following [31,32]. Hardness: the highest force peak of the first compression cycle (N). Slope: expressed as N/s, from the start of the curve to the maximum peak at the first compression cycle. It can be referred to as slope to hardness; as the slope becomes higher, the material might have less tendency to deform before fracture. Springiness: ratio between the distances the sample was compressed in the second downstroke divided by the first downstroke. Cohesiveness: ratio of the areas under the curve of the second compression cycle to the first compression cycle. Chewiness is calculated as cohesiveness × hardness × springiness (N). Resilience: ratio between the area under the curve of the withdrawal divided by the area under the curve in the downstroke, both in the first compression cycle.

### 2.6. Cheese Sensory Analysis

Sensory evaluation was carried out with seven trained assessors aged between 35 and 60 years (three men and four women). Informed consent was obtained from all subjects involved in the study. A discontinuous seven-point scale was used for texture, flavour, paste appearance, and paste holes, where 1 was the lower score, and 7 was the best score, as required for quality control of Idiazabal PDO cheese. Scores lower than 4 indicated that cheeses presented defects, and assessors were asked to identify which defects were perceived. Scores from 4 to 6 were marked when there were not defects, but the sensory characteristics were not totally appropriate [33].

Nine training sessions were conducted (around 90 min each). Four panellists belonged to the PDO Idiazabal official sensory panel, and three assessors had previous experience in sensory analysis in sheep-milk cheeses [34]. The first three training sessions were addressed to the assessors who did not belong to the PDO Idiazabal official sensory panel. All the assessors attended to the onward sessions. In the next four sessions, references were presented together with cheese samples. In the last two training sessions, the assessors evaluated cheese samples without references in order to harmonize results within the panel [33].

Sensory assessments were conducted in individual booths at the sensory laboratory, which complied with ISO 8589 standard [35]. Cheeses for each session were tempered at 17 ± 2 °C and presented rind-free and cut into parallelograms of 1.5 cm × 1.5 cm × 5 cm, and samples were randomly presented coded with a three-digit number obtained from Fizz software 2.40H (Biosystemes, Couternon, France). Low-mineralization water and Granny Smith apples were used to remove aftertaste between samples. Subsequently, whole wedges were randomly presented and identified with different three-digit numbers from Fizz software to score paste appearance and holes. In each session, assessors analysed wedge samples packaged in the six different treatments. A replicate of each session was conducted within the same day with a half-hour break and randomly presented with different coded numbers.

### 2.7. Data Treatment and Statistical Analysis

SPSS IBM Statistics software version 26.0 (New York, NY, USA) was used for statistical analysis (SPSS INC., Chicago, IL, USA). Two-way analysis of variance (ANOVA) was used to determine the significant differences in headspace and physicochemical and colour parameters from the different packaging treatments over the study period using packaging treatment and storage time as fixed factors. Subsequently, the Tukey’s test was applied to pairwise comparisons between cheeses packaged under the different treatments and on each sampling day separately. Kruskal–Wallis H test was used to check for possible significant differences between storage condition and storage time, regarding instrumental texture and sensory parameters. A stepwise discriminant analysis was applied to physicochemical, instrumental colour, texture profile, and sensory parameters to classify cheese samples from the different packaging methods considering all MAP samples as a unique group. Statistical significance was declared at *p* ≤ 0.05.

## 3. Results and Discussion

### 3.1. Headspace Gas Composition

The headspace of cheese wedges was analysed for O_2_ and CO_2_ concentration except for the vacuum-packed samples. On day 56, MAP4 pouches were totally collapsed, and the measurement was not carried out.

#### 3.1.1. O_2_ Concentration

**Figure 2:**
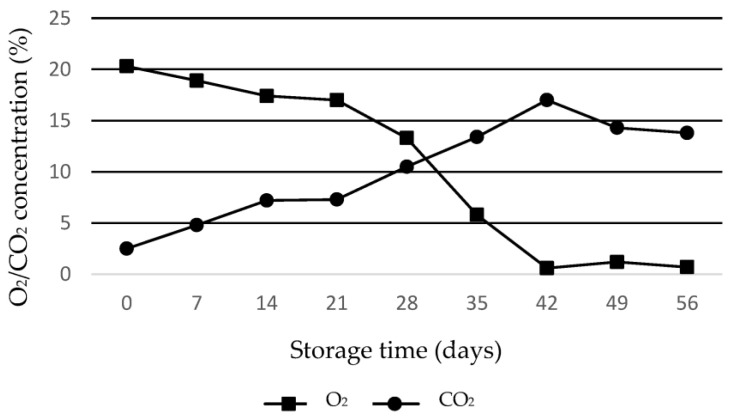
Evolution of O_2_ and CO_2_ concentrations of cheese wedges stored for eight weeks in air-packaging treatment.

In the air-packaging treatment, O_2_ concentration decreased from day 0 (20.3%) to day 56 (0.7%), resulting in a total reduction of 96.4%. From day 42, it remained constant with 0.5–0.7% (Figure 2). Film permeability, aerobic microorganisms’ metabolism, oxidative and enzymatic reactions involving oxygen, and cheese respiration could cause a progressive decrease in O_2_ concentration in the air-packaged wedges [10,36,37]. A decrease in the O_2_ concentration occurred in Domiati cheese packaged in air during cold storage, with levels from 19.9% to 0.2%, although very high barrier film was used for the samples packaging [38].

In the MAP cheese wedges, O_2_ concentration at the beginning of the storage time was residual (±0.4%), and it did not change during storage. Steady-state conditions between microbial respiration rate and O_2_ permeation through packaging material could explain this result [36], indicating non-presence of failures in the packaging [39]. According to Garabal et al. [12], there were no differences (*p* > 0.05) in O_2_ content among different MAP atmospheres of packaged cheeses, with mean values close to 0.2%. These concentrations were similar for Samso cheese [10] and Havarti cheese stored with 20 to 100% CO_2_ modified atmospheres [40].

#### 3.1.2. CO_2_ Concentration

In air-packaging treatment, there was a progressive and very pronounced increase of CO_2_ (2.9 times more) in the first 14 days (Figure 2; Table 1). After 56 days, the CO_2_ concentration increased 5.6 times. This progressive increase could be due to the gas permeability through the packaging material and the microbial growth in the cheese matrix.

Increases in CO_2_ concentration were reported for semi-hard and hard cheeses packed under MAP [11,40]. Several authors related this effect to microbial growth and cheese ripening, together with some oxidative and enzymatic reactions [11,37,41]. In the present study, in the air-packaged wedges after 28 storage days, CO_2_ concentration was increased 4.3 times, whereas that of O_2_ decreased 1.5 times (Figure 2). These data agreed with those reported for MAP-packaged Domiati cheeses. The increase of CO_2_ concentration in the gas headspace might be mainly associated with O_2_ consumption by microorganisms [38].

In MAP1, MAP2, and MAP3 treatments (Table 1), a progressive decrease of the CO_2_ concentration reaching a mean value of 39% (MAP1: 30.36%, MAP2: 44.23%, and MAP3: 43.14%) was observed at the end of the storage period. These results could be explained by the gas dissolution in the cheese matrix. Several authors pointed out that the low CO_2_ concentrations detected in headspaces may be attributed to its dissolution in the cheese matrix, its consumption by anaerobic microorganisms, or by CO_2_ loss through the barrier film [10,11,37,41]. Solomakos et al. [42] observed no important changes on CO_2_ content in the headspace of cheese stored during 85 days under 50/50% CO_2_/N_2_ (*v/v*) MAP conditions. For MAP4, CO_2_ concentration did not change until day 49 (Table 1). However, from day 28, some pouches started to collapse and on day 56 were totally collapsed, so the measurement could not be carried out. The absence of variation in the proportion of CO_2_ in the latter treatment is due to the fact that it is the only gas present in the package. The progressive decrease of the volume inside the pouches until the collapse at the end of storage may be related to the fact that it is a raw-milk cheese with a higher number of lactic acid bacteria (LAB) and/or anaerobic microorganisms that can consume this gas. Both the consumption of gas by LAB and/or the dissolution of CO_2_ in the cheese can increase the acidity, which was reflected in the results of the sensory analysis with the appearance of acid off-flavours on day 56.

### 3.2. Physicochemical Analysis of Cheeses

None of the physicochemical parameters measured in cheese wedges showed significant differences (*p* ≤ 0.05) for both the packaging treatment and storage time or the interaction between them. Thus, cheese wedges had mean values of 0.39 ± 0.43% for weight loss, 5.00 ± 0.06 for pH, 1.22 ± 0.07 g lactic acid/100 g cheese for titratable acidity, 66.13 ± 0.54% for dry matter, 24.68 ± 0.34% for protein, and 35.50 ± 0.53% for fat.

The percentage of weight loss percentage of cheese wedges during storage was not significant (*p* > 0.05) for all packaging treatments, with the mean value being 0.39 ± 0.43%. The plastic material used for packaging prevented dehydration and weight loss of cheese wedges [12,22]. This was consistent with the findings of different MAP gas mixtures that did not significantly (*p* > 0.05) influence weight losses in ripened cheeses packaged under MAP [11,37,43]. Favati et al. [17] reported weight losses of 0.15% in Provolone cheese.

The possible dissolution of CO_2_ in MAP-packaged cheeses matrix mentioned before did not affect pH value, and it remained stable throughout the storage. Other studies observed a similar behaviour for pH values in the case of other cheese types packaged under MAP conditions during refrigerated storage (ranges 4.7–4.8 for aged white cheese) [41]. Solomakos et al. [42] observed a pH decrease of air (from 5.52 to 5.10) and MAP (from 5.52 to 4.95) cheeses during storage at 10 °C, probably due to further activity and growth of LAB as compared to a lower microbial growth at 4 °C. The presence of CO_2_ in the headspaces was expected to cause a decrease in pH and an increase in the acidity of the cheese samples because of CO_2_ dissolution occurring at low temperature in cheese and the formation of carbonic acid [22,24]. Provolone samples packaged with 100% CO_2_ at 4 °C presented higher acidity, free fatty acids, and free amino acids contents than other gas mixtures [17]. However, other authors indicated that CO_2_ is mainly absorbed on food surface, which may lead to acidification of some spots along the surface rather than in the matrix [44]. Pintado and Malcata [45] reported that storing cheese above refrigeration temperatures (12 or 18 °C) resulted in a very strong pH decrease in the cheeses. These results confirm that temperature control during storage is important. On the other hand, an increase in pH (from 5.25 to 5.40 in 50% CO_2_ MAP) during storage can be due to proteolysis and associated formation of amines and ammonium [12]. It has been reported that after 45 storage days, the total amount of free amino acids in cheeses was approximately two times higher than that observed at the beginning of the process, suggesting a high rate of proteolysis during storing. At the same time, microbial degradation could be favoured by low concentrations of O_2_ in MAP packaging. A lack of any effect of the CO_2_ on cheese proteolysis during storage was reported by Alves et al. [46]. Gonzalez-Fandos et al. [22] associated increased proteolysis in vacuum-packaged Cameros cheese to the higher counts of microorganisms.

### 3.3. Instrumental Colour Parameters

None of the colour parameters showed significant differences (*p* > 0.05) for either storage time or the interaction between packaging treatment and storage time. The colour parameters L* and Zi did not show significant differences (*p* > 0.05) over time in any of the packaging treatments, while a*, b*, and Yi parameters showed differences (*p* ≤ 0.01) for the packaging treatment (Table 2). In particular, a* parameter was able to significantly distinguish air-packaged cheeses (mean value −3.52) from the rest of the treatments (mean value −2.68).

The only significant difference (*p* ≤ 0.05) over the storage period was for a* in the air-packaging treatment, going from −2.96 to −3.16, from 0 to day 56. This change was sharper the first 14 days (−3.82), and then, this value was practically maintained until the end of the storage period. The rest of the packaging treatments showed no significant differences (*p* > 0.05) over time.

Colour parameters b* and Yi showed significant differences (*p* ≤ 0.05) over time only in MAP1 treatment. The rest of the packaging treatments showed no differences (*p* > 0.05) over time. Comparing the treatments with each other, the air- and vacuum-packed cheeses (mean values for both treatments were 13.19 for b* and 22.98 for Yi) were similar, while MAP treatments (mean values for all four treatments were 12.08 for b* and 21.11 for Yi) were grouped together. The parameters Yi and Zi did not seem to discriminate more than L*, a*, and b*. A similar trend was found for the colour parameter, L*, during the first two storing months in a blue cheese [47] and Crottin-de-Chavignol-type goat cheese [19]. In portioned Canestrato pugliese cheese, vacuum and MAP might stabilize cheese colour during storage [48]. Low O_2_ transmission rates and low residual O_2_ levels in the headspace and dissolved in the cheese can help to avoid photo-oxidation of the food matrix. Trobetas et al. [11] observed a gradual discoloration, L* and b* values decreased, and a* value increased in Graviera hard cheeses packed under MAP and exposed to light at 4 °C, which was related in part to riboflavin degradation induced by light. The values for a* and b* of the samples stored in dark remained constant. Retinol and xanthophyll have been detected in low concentrations in sheep and goat milk but not β-carotene [49].

Favati et al. [17] did not detect differences in the colour parameter Yi for cow’s milk cheese packaged in portions at different CO_2_ concentrations, as reported Romani et al. [50]. Avila et al. [51] described that the increase in parameters a* and b* might be mainly due to the cheese concentration components coming from dehydration throughout ripening.

### 3.4. Texture Profile Analysis

**Figure 3:**
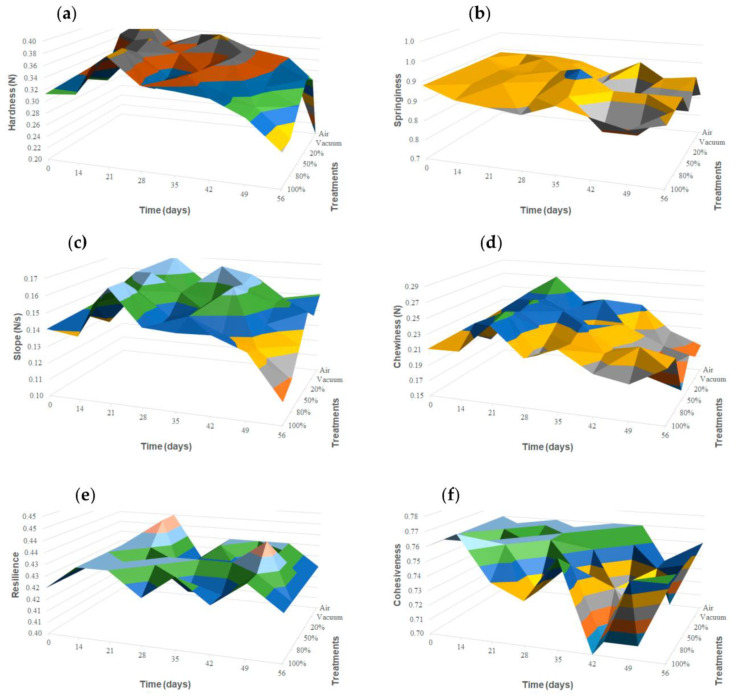
Three-dimensional surface chart for mean values of each parameters obtained in the texture profile analysis (TPA). Each graph represents a texture parameter from the TPA: (**a**) hardness, (**b**) springiness, (**c**) slope to hardness, (**d**) chewiness, (**e**) resilience, and (**f**) cohesiveness. Texture parameters are represented with respect to time (0 to 56 days) for each storage condition (air, vacuum, and increasing CO_2_ percentages from 20% to 100%).

Storage brought a significant initial increase (*p* ≤ 0.05) in some instrumental texture parameters within the first two weeks of storage (Figure 3). This was observed for hardness, slope, and chewiness in packaging treatments of air, vacuum, and MAP1 (Table S1). For hardness, this difference was also observed in MAP2-packaged cheese wedges. For further CO_2_ concentrations, this change was not noticeable. Along the eight-week storage, values tended to decrease, getting closer to those registered initially at day 0 when approaching the last storage stages (Figure 3).

Statistical analysis showed significant (*p* ≤ 0.05) changes along time in all the packaging treatments for hardness, slope, chewiness, and resilience. Atallah et al. [38] described an initial increase in cohesiveness, springiness, and chewiness that decreased from day 30 onwards. They concluded that this depended on the type of milk, production methods, and other processing conditions. Costa et al. [37] reported an increase in hardness during the first 20 storage days for ripened cheeses and related it to the change in moisture con-tent. From that day on, they observed a reduction in instrumental hardness as well, which they attributed to detrimental phenomena and moulds growth, according to the literature. Changes in moisture content were not detected, as happened in most of the physicochemical parameters studied. However, initial changes were reported in gas balances and colour a* parameters, especially for air-storage samples, which could be related to initial increase in the values of some texture attributes already described. CO_2_ dissolution in the cheese matrix might have prevented this effect happening from 20/80% CO_2_/N_2_ (*v/v*) on, as CO_2_ has proven to maintain physical, nutritional, and organoleptic features and to improve cheese microstructure through component interactions [52]. Nevertheless, microbial growth in two weeks’ time might have been enough to cause a decrease in texture values after the initial increase in air, vacuum, and MP1 samples and during storage for the other MAP treatments.

Generalized significant (*p* ≤ 0.05) changes in cheese texture between packaging treatments were only perceived for hardness and chewiness. Hardness showed a significant decrease along time, with the lowest values from 42 days on. This decrease was higher for those MAP treatments with highest CO_2_ concentration (>50%). Significant changes among treatments were also registered for chewiness in all the storage time points except for 21 and 35 days. Chewiness values tended to decrease along time for air-packaged, vacuum, MAP1, and MAP2 treatments, while the last storage stages showed similar values for MAP3 compared to storage at 14 days (0.237 ± 0.049 N to 0.217 ± 0.060 N) and higher for MAP4 (0.212 ± 0.053 N to 0.218 ± 0.032 N) compared to storage at 14 days. Some previous studies had different results, probably due to differences in milk characteristics, ripening time, and storage conditions. Kirkin et al. [41] showed that hardness was higher in 75/25% CO_2_/N_2_ MAP (*v/v*) compared with the vacuum packaging considering the overall mean during the entire storage period. Favati et al. [17] reported the lowest shear force values for vacuum-packed Provolone cheeses compared to CO_2_-containing atmospheres (10–100%).

### 3.5. Sensory Properties of Cheeses

On days 49 and 56, it was not possible to analyse texture and flavour of air-packaged cheese wedges due to the growth of mould spots in the paste (Table 3). The texture of air- packaged cheeses was dry and lumpy after 21 storage days, and flavour scores were below acceptance from day 35 onwards with mouldy notes.

Short storage times have been described for cheeses kept in air on account of mould growth [19,22,44,53]. Vacuum-packaged cheese wedges were acceptable at all storage times, with slight differences in texture and flavour. Other vacuum-packaged cheeses were softer and more elastic due to possible fat migration. Garabal et al. [12] and Romani et al. [13,50] detected an increase in acidity with this preservation method.

Among the MAP treatments, only MAP1 samples showed differences (*p* ≤ 0.05) between day 0 and 56 for texture, but on day 49, assessors signalled wet-mouldy flavours, and cheeses had an assessment below the limit in flavour parameter on the last storage day. Esmer et al. [19] reported that cheese packaged at low CO_2_ concentration quality was randomly affected on 42 days, and Garabal et al. [12] described the cheeses as friable and grainy. From a safety pointy of view, this low CO_2_ concentration was at the limit for microorganisms’ inhibition, which corresponds to the presence of mouldy flavours in this study [9].

Sensory texture and flavour scores for MAP4 were below the minimum acceptance mark at 56 days (Table 3). Assessors indicated texture defects as lumpy and fracturable, and crystals were perceived on chewing. Juric et al. [10] found dry and crumbly texture in cheeses packed with high CO_2_ concentration. Agarwal et al. [54] described calcium lactate crystals in Cheddar cheese packages with 100/0% CO_2_/N_2_ (*v/v*) after four storage weeks. A possible reason for crystal formation is the cheese’s superficial drying, which may favour the onset of calcium lactate crystallization. Assessors highlighted acid, rancid, and pungent notes as off-flavours. These off-flavours may be the result of CO_2_ solubilisation in the cheese matrix since the pouches, as discussed above in Section 3.1.2, were collapsed. Romani et al. [50] and Gonzalez-Fandos et al. [22] observed that high CO_2_ atmosphere produced great flavour variations, and they attributed this to the solubilisation of CO_2_ in the cheese matrix, which produced acidity, and the storage was shortened compared to other MAP conditions [53]. MAP2 and MAP3 showed no significant changes for texture and flavour (*p* > 0.05) during 56 storage days. Several authors pointed out that atmosphere close to 50/50% CO_2_/N_2_ (*v/v*) is the best for preserving cheese flavour [11,13,22,41,44,50,53,55].

For paste appearance, the panel scored vacuum- and air-packaged cheeses as unacceptable from day 14 and 28 onwards, respectively (Table 4). Vacuum packaging on day 14 showed a greasy, plastic-like paste appearance, with very small white spots (Figure S1). At the end of the storage period, non-homogeneous colourings appeared with white areas and pronounced marks caused by packaging shrinkage. This anomalous look was also observed in Parmegiano Reggiano cheese, and it was described as the oil-dropping phenomena, in which there is a migration of fat to the surface due to lipid hydrolysis [13,50]. From day 28 onwards, air-packaged samples exhibited a non-homogeneous appearance and obtained a score below 4. On day 42, batches presented spot moulds and small crystals (well-defined, round, white marks with relief) on the paste. During the last two storage days, moulds were more noticeable. Costa et al. [37] and Atallah et al. [38] indicated that appearance was the limiting factor in air-packaged cheeses. MAP4 scored below the limit on days 21 and 49. On day 21, they presented a whiter appearance and small crystals and, on day 49, small black spots in the paste, non-homogeneous colour, and small crystals. The presence of crystals corresponded to the perception described as a defect in texture at the end of storage. At high CO_2_ concentration, free ionic calcium combines with lactate through a mechanisms involving carbonic acid, resulting in calcium lactate crystals [56]. Costa et al. [37] found a variation in surface colour with crystals at these conditions, also possibly due to calcium lactate formation that may result from growth of non-starter LAB. MAP1 and MAP2 samples remained above the acceptance limit value at all times, and no differences (*p* > 0.05) were found for paste appearance in MAP3 cheeses throughout storage (Table 4).

According to Idiazabal PDO specifications, cheese must present few small irregular holes homogeneously distributed throughout the cheese paste [5]. Vacuum-packed wedges showed a significant (*p* ≤ 0.05) degradation from day 14 onwards (Table 4), and holes started to occlude due to packaging pressure. This fact is aggravated over time, and after two storage months, the cheese paste had sinkholes where holes were initially located. The holes of air-packaged cheeses were scored lower than the MAP-preserved cheeses. This could be due to the intrinsic variation in the cheese holes distribution. Cheeses stored under MAP conditions showed small variations and were always above the limit of the disqualification score. The best-rated cheeses were MAP2 and MAP3. In the scientific literature, there are no remarks on the behaviour of the natural paste holes in MAP-packaged and vacuum-packaged cheese.

### 3.6. Discriminant Analysis

**Figure 4:**
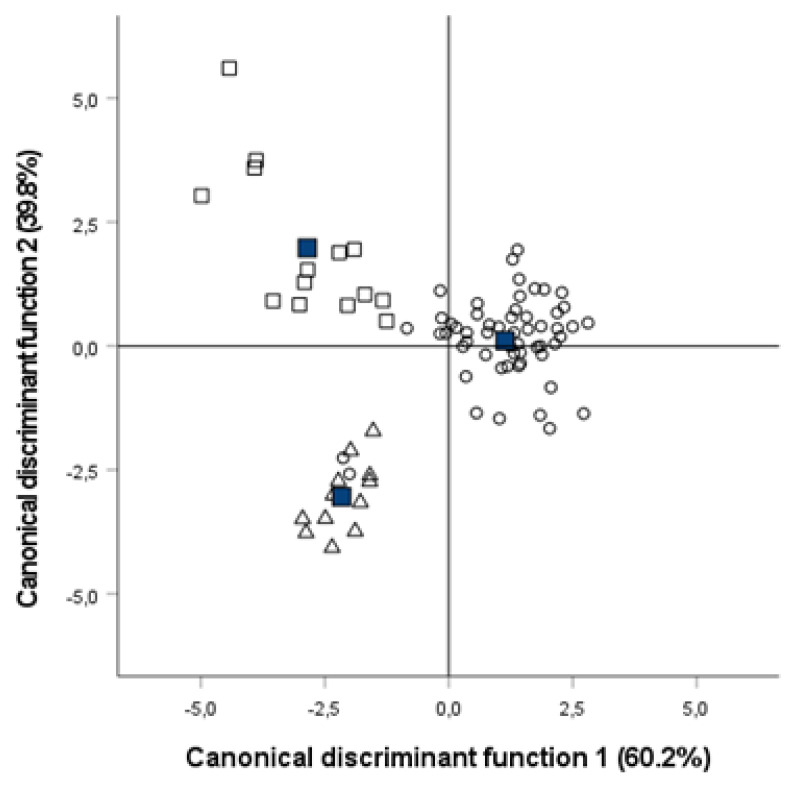
Graph for the two canonical discriminant functions corresponding to the stepwise discriminant analysis on compositional, instrumental colour, and texture and sensory parameters of packaged cheese wedges stored for eight weeks at different atmosphere treatments. Air, ∆; vacuum, □; modified atmosphere packaging, ○.

Discriminant analysis was applied to cheese physicochemical variables, instrumental colour, and texture and sensory parameters to classify cheese samples according to the packaging treatment applied considering MAP conditions as a unique group and irrespective of the storage time. Figure 4 shows the cheese sample distribution in the graph displaying the two canonical discriminant functions.

In general, results showed that 97.6% of the samples were correctly classified into their corresponding treatment group (air-packaged, vacuum-packaged, and MAP). The discriminant variables with higher correlation with canonical functions in the structure matrix were the sensory parameters paste appearance and holes, flavour, the colour parameters b* and a*, and the instrumental texture parameter slope. The cross-validation method used for sample classification reported that all air-packaged samples were correctly grouped and that the 96.4% and 92.9% of the MAP and vacuum-cheese samples, respectively, were correctly assigned.

## 4. Conclusions

In this study of semi-hard raw-milk cheeses, different gas-packaging treatments were tested (air, vacuum, and MAP). Experimental results highlighted that none of the treatments changed the physicochemical composition. There were no significant physicochemical changes during the storage period studied. The characteristics with the greatest weight in the packaging treatment differentiation were paste appearance and holes, flavour, a*and b* colour parameters, and slope texture parameter. Sensory analysis based on Idiazabal cheeses quality control requirements was decisive to select the best packaging conditions.

MAP-preserved cheese wedges’ quality was better than that of air or vacuum packaging. The absence of oxygen in MAP and vacuum conditions contributed to colour stability in view of the changes observed in the parameter a* in air-packaged cheese wedges. The air-packed atmosphere was not the best option for storing cheese wedges since they had a short shelf life caused by gas changes in the atmosphere. Together with the colour, the limiting factor was the presence of moulds, giving mouldy flavours. The traditional vacuum-packaging was not a valid option either; although many parameters were not affected, sensory appearance was low-rated in very early stages of storage, rendering these cheeses unacceptable.

Regarding MAP treatments, very low concentrations of CO_2_ were not sufficient to inhibit the growth of microorganisms, as mouldy flavours were observed, and texture was compromised. For ≥50/50% CO_2_/N_2_ (*v/v*) atmosphere, texture parameters (hardness, chewiness, and slope) remained stable, while changes were significant for wedges packaged under lower CO_2_ concentration. However, at 100% CO_2_ concentration, the pouches collapsed, sensory texture declined, and off-flavours appeared. For a storage period of two months, mixtures between 50/50 and 80/20% CO_2_/N_2_ (*v/v*) resulted as the most useful techniques to ensure sensory quality for these cheeses.

These results can be of great interest for dairy farms, cheese industries, distribution chains, retail points, and consumers, as these preservation techniques can improve the cheese storage period. Given the inherent interest in the sector, the behaviour of sustainable materials (recyclable or biodegradable) in selected packaging options could be further explored.

## Figures and Tables

**Table 1:** Mean, standard deviation, and significance level of ANOVA for CO_2_ concentration (%) in the headspace of packaged cheese wedges stored for eight weeks at different atmosphere treatments.

Day	Air	MAP1	MAP2	MAP3	MAP4
0	2.45 ± 0.78 ^d^	23.98 ± 0.67 ^a^	52.90 ± 0.78 ^a^	83.80 ± 0.35 ^a^	100.08 ± 0.04 ^a^
14	7.20 ± 1.56 ^cd^	19.20 ± 0.99 ^ab^	44.75 ± 0.78 ^ab^	76.80 ± 0.99 ^ab^	100.00 ± 0.00 ^a^
21	7.25 ± 0.78 ^cd^	17.70 ± 0.57 ^b^	40.60 ± 1.41 ^bc^	73.65 ± 2.62 ^ab^	99.95 ± 0.07 ^a^
28	10.49 ± 0.69 ^bc^	18.30 ± 1.56 ^ab^	40.50 ± 1.98 ^bc^	70.40 ± 6.36 ^ab^	97.95 ± 2.90 ^a^
35	13.35 ± 4.03 ^abc^	17.05 ± 0.21 ^b^	37.53 ± 2.02 ^bcd^	64.20 ± 8.34 ^ab^	94.65 ± 7.42 ^a^
42	17.00 ± 0.57 ^a^	17.95 ± 2.05 ^ab^	38.35 ± 4.45 ^bcd^	66.00 ± 9.90 ^ab^	93.80 ± 8.77 ^a^
49	14.25 ± 0.49 ^ab^	17.15 ± 1.34 ^b^	33.90 ± 2.40 ^cd^	59.80 ± 14.85 ^ab^	99.10 ± 0.00 ^a^
56	13.80 ± 0.57 ^ab^	16.70 ± 2.97 ^b^	29.50 ± 4.38 ^d^	47.65 ± 14.07 ^b^	-

MAP1, 20/80% CO_2_/N_2_ (*v*/*v*); MAP2, 50/50% CO_2_/N_2_ (*v*/*v*); MAP3, 80/20% CO_2_/N_2_ (*v/v*); MAP4, 100/0% CO_2_/N_2_ (*v/v*). Different letters (a–d) in the same column indicate significant differences (*p* ≤ 0.05) during storage for each packaging treatment.

**Table 2:** Mean, standard deviation, and significance level of ANOVA for packaging treatment, storage time, and interaction between them for the colour parameters L*, a*, b*, Yi, and Zi.

	L*	a*	b*	Yi	Zi
Packaging treatment	ns	**	**	**	ns
Storage time	ns	ns	ns	ns	ns
Interaction	ns	ns	ns	ns	ns
x ± SD	81.27 ± 2.22	−2.80 ± 0.47	12.27 ± 0.93	21.54 ± 1.56	47.04 ± 3.37

L*, lightness; a*, redness; b*, yellowness; yellow index Yi, 142.86 b*/L*; yellowness index Zi, 100(L* + 16/116)–(b*/200)^3^; ** *p* ≤ 0.01; ns, not-significant differences.

**Table 3:** Sensory texture and flavour: mean, standard deviation, and significance level of Kruskal–Wallis H statistics treatment of packaged cheese wedges stored for eight weeks at different atmosphere treatments.

Texture						
Day	Air	Vacuum	MAP1	MAP2	MAP3	MAP4
0	5.36 ± 0.63 ^a1^	5.36 ± 0.63 ^a1^	5.36 ± 0.63 ^a1^	5.36 ± 0.63 ^a1^	5.36 ± 0.63 ^a1^	5.36 ± 0.63 ^a1^
14	5.07 ± 0.92 ^a12^	5.29 ± 0.9 ^a12^	5.14 ± 0.86 ^a12^	5.18 ± 0.77 ^a1^	5.07 ± 0.83 ^a1^	4.75 ± 0.75 ^a12^
21	4.84 ± 0.89 ^a12^	5.00 ± 0.68 ^a12^	5.07 ± 1.00 ^a12^	4.79 ± 0.98 ^a1^	4.43 ± 0.51 ^a1^	4.71 ± 0.83 ^a12^
28	4.50 ± 0.76 ^a12^	4.79 ± 0.80 ^a12^	4.71 ± 0.73 ^a12^	4.86 ± 0.77 ^a1^	5.00 ± 0.88 ^a1^	4.64 ± 0.74 ^a12^
35	4.21 ± 0.43 ^a2^	4.64 ± 0.74 ^a12^	4.50 ± 0.65 ^a12^	4.77 ± 0.70 ^a1^	4.86 ± 0.86 ^a1^	4.14 ± 0.66 ^a2^
42	4.50 ± 0.52 ^a12^	4.92 ± 0.83 ^a12^	4.79 ± 0.58 ^a12^	4.93 ± 0.88 ^a1^	5.14 ± 0.77 ^a1^	4.50 ± 0.66 ^a12^
49	-	4.79 ± 0.80 ^a12^	4.50 ± 0.85 ^a12^	5.21 ± 0.89 ^a1^	4.79 ± 0.56 ^a1^	4.21 ± 0.43 ^a12^
56	-	4.36 ± 0.50 ^a2^	4.43 ± 0.51 ^ab2^	4.64 ± 0.74 ^ab1^	4.86 ± 0.66 ^a1^	3.86 ± 0.77 ^b2^
**Flavour**						
0	5.50 ± 0.52 ^a1^	5.50 ± 0.5 ^a1^	5.50 ± 0.52 ^a1^	5.50 ± 0.52 ^a1^	5.50 ± 0.52 ^a1^	5.50 ± 0.52 ^a1^
14	5.07 ± 0.83 ^a12^	5.14 ± 0.66 ^a12^	5.07 ± 0.83 ^a1^	5.21 ± 0.58 ^a1^	5.07 ± 0.73 ^a1^	5.04 ± 0.80 ^a12^
21	4.43 ± 0.65 ^a234^	4.93 ± 0.9 ^a23^	4.93 ± 0.73 ^a1^	5.00 ± 0.78 ^a1^	4.86 ± 0.86 ^a1^	4.29 ± 0.73 ^a23^
28	4.23 ± 0.73 ^a234^	4.29 ± 0.61 ^a3^	4.64 ± 0.84 ^a123^	4.86 ± 0.77 ^a1^	4.79 ± 0.89 ^a1^	4.43 ± 0.76 ^a23^
35	3.93 ± 0.47 ^bc34^	4.50 ± 0.6 ^ab23^	4.50 ±0.85 ^ab123^	4.57 ± 0.7 ^ab1^	4.71 ± 0.73 ^ab1^	4.14 ± 0.6 ^bc23^
42	3.36 ± 1.00 ^c4^	5.21 ± 0.70 ^ab12^	4.86 ± 0.86 ^ab12^	4.93 ± 0.83 ^ab1^	5.36 ± 0.63 ^a1^	4.29 ± 0.92 ^bc23^
49	-	4.79 ± 0.80 ^a12^	4.50 ± 0.85 ^a12^	5.21 ± 0.89 ^a1^	4.79 ± 0.56 ^a1^	4.21 ± 0.43 ^a12^
56	-	4.50 ± 0.94 ^ab23^	3.71 ± 0.71 ^b3^	4.57 ± 0.76 ^a1^	4.79 ± 0.43 ^a1^	3.71 ± 0.91 ^ab3^

MAP1, 20/80% CO_2_/N_2_ (*v/v*); MAP2, 50/50% CO_2_/N_2_ (*v/v*); MAP3, 80/20% CO_2_/N_2_ (*v/v*); MAP4, 100/0% CO_2_/N_2_ (*v/v*). Different letters (a–c) in the same row indicate significant differences (*p* ≤ 0.05) between the different packaging conditions on that day. Different numbers (1–4) in the same column indicate significant differences (*p* ≤ 0.05) during storage for each packaging condition.

**Table 4:** Sensory paste appearance and holes: mean, standard deviation, and significance level of Kruskal–Wallis H statistics treatment of packaged cheese wedges stored for eight weeks at different atmosphere treatments.

Paste Appearance						
Day	Air	Vacuum	MAP1	MAP2	MAP3	MAP4
0	5.43 ± 0.65 ^a1^	5.43 ± 0.65 ^a1^	5.43 ± 0.65 ^a1^	5.43 ± 0.65 ^a1^	5.43 ± 0.65 ^a1^	5.43 ± 0.65 ^a1^
14	4.71 ± 1.07 ^ab12^	3.86 ± 0.66 ^b12^	5.14 ± 0.66 ^a12^	5.21 ± 0.89 ^a12^	4.64 ± 0.63 ^ab1^	5.27 ± 0.83 ^a12^
21	4.21 ± 0.70 ^ab12^	3.36 ± 0.74 ^b2^	4.36 ± 1.01 ^ab2^	4.64 ± 0.84 ^a123^	4.50 ± 1.02 ^a1^	3.93 ± 0.73 ^ab23^
28	3.71 ± 0.73 ^bc23^	3.14 ± 0.36 ^c23^	4.43 ± 0.85 ^ab12^	4.36 ± 0.74 ^ab23^	4.93 ± 0.83 ^a1^	4.21 ± 0.98 ^ab12^
35	3.36 ± 0.50 ^b234^	3.21 ± 0.58 ^b23^	4.36 ± 0.84 ^a2^	4.21 ± 0.70 ^a23^	4.79 ± 0.80 ^a1^	4.00 ± 0.78 ^ab23^
42	1.79 ± 1.05 ^b4^	2.78 ± 0.43 ^b234^	4.71 ± 0.91 ^a12^	4.57 ± 0.75 ^a123^	5.29 ± 0.73 ^a1^	4.29 ± 0.73 ^a12^
49	2.14 ± 0.66 ^b34^	2.21 ± 0.43 ^b34^	4.86 ± 0.53 ^a12^	4.50 ± 0.52 ^a123^	4.93 ± 0.73 ^a1^	2.86 ± 0.53 ^b3^
56	1.64 ± 0.74 ^b4^	1.64 ± 0.63 ^b4^	4.29 ± 0.61 ^a2^	4.00 ± 0.78 ^a3^	4.50 ± 0.65 ^a1^	4.22 ± 0.80 ^a12^
**Holes**						
0	5.64 ± 0.50 ^a1^	5.64 ± 0.50 ^a1^	5.64 ± 0.50 ^a1^	5.64 ± 0.50 ^a1^	5.64 ± 0.50 ^a1^	5.64 ± 0.50 ^a1^
14	4.36 ± 1.08 ^ab2^	4.00 ± 0.78 ^b12^	5.21 ± 1.31 ^ab12^	5.36 ± 0.74 ^ab1^	5.36 ± 0.50 ^ab12^	5.79 ± 0.70 ^a1^
21	4.14 ± 0.36 ^ab2^	3.35 ± 0.63 ^b23^	4.14 ± 0.66 ^ab3^	4.43 ± 0.51 ^a3^	4.71 ± 0.47 ^a2^	4.21 ± 0.43 ^a2^
28	4.29 ± 0.72 ^bc2^	3.50 ± 0.65 ^c23^	4.64 ±0.84 ^ab123^	5.50 ± 0.52 ^a1^	5.5 ± 0.52 ^a1^	4.86 ± 0.77 ^ab12^
35	4.00 ± 0.55 ^bc2^	3.28 ± 0.61 ^c23^	4.64 ±0.84 ^ab123^	5.21 ± 0.70 ^a123^	5.21 ± 0.80 ^a12^	4.50 ± 0.52 ^ab2^
42	3.93 ± 1.54 ^bc23^	2.71 ± 0.47 ^bc34^	5.43 ± 0.76 ^a1^	5.50 ± 0.52 ^a1^	5.64 ± 0.63 ^a1^	4.64 ± 0.75 ^ab2^
49	2.29 ± 0.47 ^c3^	2.70 ± 0.83 ^bc34^	4.36 ± 0.50 ^ab23^	4.50 ± 0.52 ^a23^	5.14 ± 0.66 ^a12^	4.07 ± 0.73 ^ab2^
56	4.14 ± 1.02 ^b2^	1.00 ± 0.00 ^c4^	5.14 ± 0.66 ^ab12^	5.36 ± 0.50 ^a12^	5.00 ± 0.55 ^ab12^	4.64 ± 0.63 ^ab2^

MAP1, 20/80% CO_2_/N_2_ (*v/v*); MAP2, 50/50% CO_2_/N_2_ (*v/v*); MAP3, 80/20% CO_2_/N_2_ (*v/v*); MAP4, 100/0% CO_2_/N_2_ (*v/v*). Different letters (a–c) in the same row indicate significant differences (*p* ≤ 0.05) between the different packaging conditions on that day. Different numbers (1–4) in the same column indicate significant differences (*p* ≤ 0.05) during storage for each packaging condition.

## Data Availability

Data available on request from the corresponding author.

## Supplementary Materials

The following supporting information can be downloaded at: https://www.mdpi.com/article/10.3390/foods12040849/s1, Table S1: Mean, standard deviation and significance level of Kruskal–Wallis H for texture analysis profile of packaged cheese wedges stored for eight weeks at different atmosphere treatments. Figure S1: Photograph of cheese paste with examples of white spot area in red and small crystals in blue.
