# Supplementary material for: Optimization of Modified Atmosphere Packaging for Sheep’s Milk Semi-Hard Cheese Wedges during Refrigerated Storage: Physicochemical and Sensory Properties
Source: Foods. 2023 Feb 16;12(4):849. doi: 10.3390/foods12040849 (PMC9956239; doi:10.3390/foods12040849)
Supplement: Supplementary file 1 [file foods-12-00849-s001.zip › Figure S1.pdf]

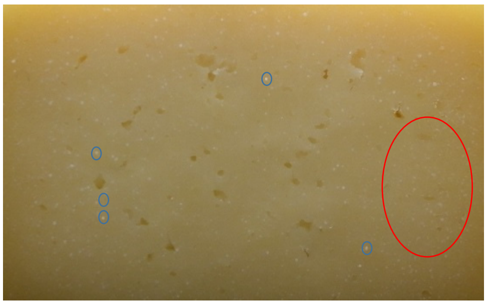

Figure S1. Photograph of cheese paste with examples of white spot area in red and small crystals in blue.
