# Supplementary material for: Optimization of Modified Atmosphere Packaging for Sheep’s Milk Semi-Hard Cheese Wedges during Refrigerated Storage: Physicochemical and Sensory Properties
Source: Foods. 2023 Feb 16;12(4):849. doi: 10.3390/foods12040849 (PMC9956239; doi:10.3390/foods12040849)
Supplement: Supplementary file 1 [file foods-12-00849-s001.zip › Table S1.pdf]

Table S1. Mean, standard deviation and significance level of Kruskal-Wallis H for Texture Analysis Profile of packaged cheese wedges stored for eight weeks at different atmosphere treatments.

Treatment	Days	Hardness (N)	Slope (N/s)	Springiness	Chewiness (N)	Resilience	Cohesiveness
	0	0.313±0.054^{3}	0.141±0.026^{3}	0.890±0.020^{1}	0.211±0.036^{3}	0.420±0.010^{2}	0.760±0.010^{1}
Air	14	0.365±0.046^{a12}	0.162±0.024^{a12}	0.890±0.030^{a1}	0.245±0.035^{a2}	0.420±0.010^{a2}	0.750±0.020^{a1}
	21	0.399±0.064^{a1}	0.176±0.028^{a1}	0.890±0.030^{ab1}	0.270±0.048^{a1}	0.440±0.010^{ab1}	0.760±0.010^{a1}
	28	0.355±0.033^{a12}	0.156±0.015^{a123}	0.880±0.030^{a1}	0.236±0.023^{a2}	0.420±0.010^{a2}	0.750±0.020^{a1}
	35	0.374±0.042^{ab12}	0.163±0.020^{ab2}	0.850±0.170^{b1}	0.224±0.072^{a2}	0.430±0.020^{a12}	0.760±0.020^{a1}
	42	0.360±0.036^{ab12}	0.158±0.019^{ab2}	0.890±0.020^{a1}	0.237±0.065^{ab12}	0.430±0.010^{a12}	0.760±0.020^{a1}
	49	0.333±0.049^{a23}	0.144±0.024^{ab23}	0.790±0.290^{a1}	0.202±0.094^{a23}	0.430±0.020^{a12}	0.700±0.190^{a1}
	56	0.341±0.052^{a23}	0.151±0.025^{a23}	0.810±0.260^{a1}	0.186±0.079^{ab3}	0.420±0.010^{a2}	0.750±0.010^{a1}
Vacuum	14	0.366±0.037^{a1}	0.162±0.022^{a1}	0.860±0.140^{a1}	0.242±0.025^{a1}	0.430±0.020^{a12}	0.760±0.020^{a1}
	21	0.355±0.055^{a1}	0.156±0.024^{a1}	0.900±0.040^{a1}	0.245±0.043^{a1}	0.440±0.020^{a1}	0.760±0.010^{a1}
	28	0.336±0.029^{ab12}	0.146±0.015^{ab}	0.890±0.030^{a1}	0.226±0.021^{ab12}	0.420±0.010^{a2}	0.760±0.020^{a1}
	35	0.386±0.064^{a1}	0.169±0.028^{a1}	0.840±0.220^{b1}	0.251±0.044^{a1}	0.430±0.010^{a12}	0.760±0.010^{a1}
	42	0.365±0.043^{ab1}	0.165±0.023^{a1}	0.770±0.320^{a1}	0.205±0.060^{b2}	0.420±0.010^{a12}	0.750±0.020^{a1}
	49	0.339±0.036^{a12}	0.151±0.018^{a23}	0.870±0.160^{a1}	0.192±0.069^{ab2}	0.430±0.020^{a12}	0.740±0.140^{a1}
	56	0.316±0.049^{a2}	0.141±0.022^{a2}	0.880±0.160^{a1}	0.181±0.044^{b2}	0.420±0.020^{a2}	0.750±0.020^{a1}
MAP1	14	0.357±0.040^{a12}	0.157±0.022^{a12}	0.880±0.020^{a1}	0.233±0.053^{ab12}	0.430±0.010^{a12}	0.760±0.010^{a1}
	21	0.382±0.038^{a1}	0.166±0.019^{a1}	0.880±0.030^{ab1}	0.253±0.048^{a1}	0.430±0.020^{ab1}	0.750±0.120^{a1}
	28	0.359±0.027^{a12}	0.154±0.013^{a12}	0.890±0.020^{a1}	0.241±0.020^{a1}	0.420±0.010^{a12}	0.760±0.020^{a1}
	35	0.345±0.043^{ab2}	0.151±0.021^{ab23}	0.880±0.160^{ab1}	0.225±0.087^{a12}	0.420±0.020^{a12}	0.750±0.020^{a1}
	42	0.369±0.056^{a12}	0.162±0.029^{a12}	0.810±0.260^{a1}	0.228±0.090^{a1}	0.420±0.020^{a12}	0.700±0.180^{a1}
	49	0.339±0.055^{a2}	0.152±0.024^{a23}	0.810±0.270^{a1}	0.186±0.085^{ab3}	0.430±0.020^{a1}	0.740±0.120^{a1}
	56	0.330±0.061^{a2}	0.148±0.030^{a23}	0.810±0.270^{a1}	0.196±0.057^{ab23}	0.420±0.010^{a2}	0.750±0.020^{a1}
MAP2	14	0.314±0.041^{bc2}	0.137±0.018^{b2}	0.880±0.040^{a2}	0.205±0.050^{b12}	0.430±0.020^{a12}	0.740±0.150^{a1}
	21	0.368±0.042^{a1}	0.158±0.020^{a1}	0.890±0.020^{ab1}	0.246±0.037^{a1}	0.420±0.030^{b12}	0.760±0.030^{a1}
	28	0.329±0.032^{b23}	0.144±0.016^{b12}	0.880±0.020^{a2}	0.220±0.023^{b2}	0.430±0.010^{a12}	0.760±0.010^{a1}
	35	0.343±0.049^{ab12}	0.150±0.021^{ab12}	0.920±0.040^{a12}	0.236±0.053^{a1}	0.430±0.020^{a12}	0.710±0.190^{a1}
	42	0.369±0.050^{ab12}	0.164±0.025^{a1}	0.870±0.140^{a12}	0.208±0.068^{c2}	0.430±0.020^{a12}	0.730±0.130^{a1}
	49	0.326±0.051^{ab23}	0.144±0.020^{ab12}	0.870±0.170^{a12}	0.176±0.076^{b2}	0.440±0.010^{a1}	0.710±0.200^{a1}
	56	0.298±0.050^{ab3}	0.133±0.024^{ab2}	0.870±0.180^{a2}	0.163±0.067^{b2}	0.420±0.020^{a2}	0.750±0.020^{a1}
MAP3	14	0.351±0.070^{ab23}	0.153±0.029^{ab12}	0.880±0.020^{a}	0.237±0.049^{ab12}	0.430±0.010^{a1}	0.760±0.010^{a1}
	21	0.386±0.055^{a1}	0.167±0.026^{a1}	0.890±0.030^{ab}	0.256±0.040^{a1}	0.430±0.020^{b12}	0.760±0.020^{a1}
	28	0.322±0.036^{b23}	0.141±0.019^{b2}	0.860±0.160^{a}	0.204±0.058^{b2}	0.430±0.020^{a12}	0.730±0.140^{a1}
	35	0.349±0.041^{ab12}	0.150±0.019^{ab12}	0.870±0.160^{ab}	0.222±0.023^{a12}	0.420±0.020^{a12}	0.750±0.020^{a1}
	42	0.326±0.051^{ab23}	0.141±0.025^{ab12}	0.790±0.280^{a}	0.214±0.106^{bc12}	0.420±0.020^{a12}	0.690±0.210^{a1}
	49	0.307±0.049^{b3}	0.135±0.024^{b23}	0.790±0.310^{a}	0.227±0.089^{ab12}	0.430±0.020^{a1}	0.680±0.220^{a1}
	56	0.261±0.042^{b3}	0.119±0.021^{b3}	0.860±0.180^{a}	0.217±0.060^{a12}	0.420±0.020^{a2}	0.750±0.020^{a1}
MAP4	14	0.317±0.057^{c12}	0.138±0.026^{ab12}	0.860±0.150^{a}	0.212±0.053^{b2}	0.430±0.020^{a1}	0.770±0.020^{a1}
	21	0.384±0.064^{a1}	0.167±0.028^{a1}	0.850±0.140^{b}	0.248±0.076^{a1}	0.430±0.020^{b1}	0.740±0.130^{a1}
	28	0.341±0.029^{b1}	0.146±0.015^{b2}	0.840±0.210^{a}	0.212±0.071^{b12}	0.420±0.010^{a12}	0.730±0.140^{a1}
	35	0.337±0.053^{bc12}	0.145±0.025^{ab12}	0.870±0.160^{ab}	0.227±0.069^{a12}	0.430±0.020^{a1}	0.760±0.020^{a1}
	42	0.330±0.050^{bc12}	0.146±0.024^{ab12}	0.850±0.190^{a}	0.202±0.077^{bc12}	0.420±0.020^{a12}	0.700±0.190^{a1}
	49	0.305±0.064^{ab23}	0.138±0.029^{b23}	0.830±0.260^{a}	0.194±0.067^{ab2}	0.430±0.020^{a12}	0.750±0.020^{a1}
	56	0.258±0.035^{b3}	0.113±0.018^{b3}	0.850±0.190^{a}	0.218±0.032^{a12}	0.420±0.020^{a12}	0.750±0.020^{a1}

MAP1: 20/80% CO_2N_2 (v/v); MAP2: 50/50% CO_2/N_2 (v/v); MAP3: 80/20% CO_2/N_2 (v/v); MAP4: 100/0% CO_2/N_2 (v/v)

Different letters (a-c) in the same column indicate significant differences ($p \leq 0.05$) between the different packaging conditions on that day

Different numbers (1-3) in the same column indicate significant differences ($p \leq 0.05$) during storage time for each packaging condition
